# G-CuP: the effect of a forced oral glucose intake on alcohol craving and mesolimbic cue reactivity in alcohol dependence—study protocol of a randomized, double-blind, placebo-controlled crossover study

**DOI:** 10.1186/s13063-022-06626-w

**Published:** 2022-08-19

**Authors:** Lea Wetzel, Madeleine Pourbaix, Alisa Riegler, Anna-Maria Pfeifer, Iris Reinhard, Sabine Hoffmann, Sabine Vollstädt-Klein, Falk Kiefer, Wolfgang Sommer, Jan Malte Bumb, Patrick Back, Anne Koopmann

**Affiliations:** 1 Medical Faculty Mannheim, Heidelberg University, Heidelberg, Germany; 2 Department of Addictive Behavior and Addiction Medicine, Central Institute of Mental Health (CIMH), Medical Faculty Mannheim/ Heidelberg University, Mannheim, Germany; 3 Feuerlein Centre on Translational Addiction Medicine (FCTS), University of Heidelberg, Heidelberg, Germany; 4 Department of Biostatistics, Central Institute of Mental Health (CIMH), Medical Faculty Mannheim/Heidelberg University, Mannheim, Germany; 5 Institute of Psychopharmacology, Central Institute of Mental Health (CIMH), Medical Faculty Mannheim/Heidelberg University, Mannheim, Germany

**Keywords:** Alcohol use disorder, Addiction, Cue reactivity, Functional magnetic resonance imaging, Appetite-regulating peptides, Neuroendocrinology, Randomized controlled trial

## Abstract

**Background:**

Multiple studies indicate that a lower plasma level of the acetylated form of the appetite-regulating hormone ghrelin and higher plasma levels of insulin lead to a reduction in subjective alcohol craving and a reduced mesolimbic cue reactivity in functional magnetic resonance imaging (fMRI) when being exposed to alcohol-associated stimuli. The ghrelin level can physiologically be reduced by the induction of stomach distension and the ingestion of glucose or lipids.

**Methods:**

A total of 108 alcohol-dependent patients aged between 18 and 65 years are examined in the randomized, double-blind, placebo-controlled crossover study. After collecting demographic and psychometric data, participants take part in an alcohol exposure session. Afterwards, the participants go through the intervention condition (oral glucose intake) and the control condition (placebo intake) in a randomized order on two examination days. Blood samples are taken repeatedly (every 10 min) during the study course on both measuring days to determine changes in acetylated and total ghrelin and insulin plasma levels. In parallel, subjective alcohol craving after the glucose or placebo intake as the primary outcome is assessed using the Alcohol Urge Questionnaire (AUQ) and a visual analog scale (VAS). To examine the mesolimbic cue reactivity as the secondary outcome, a fMRI measurement is conducted while being exposed to alcohol-related stimuli. Appropriate statistical analysis will be used for the evaluation of the outcomes.

**Discussion:**

If successful, the results of this study could offer alcohol-dependent patients a new potential option for acute short-term reduction of alcohol craving and thus prevent relapses and prolong periods of abstinence in the long term.

**Trial registration:**

German Clinical Trials Register DRKS00022419 (UTN: U1111-1278-9428). Retrospectively registered on September 15, 2020.

## Background

Alcohol dependence (AD; according to ICD-10 [1]) is an important public health problem worldwide with a mean lifetime prevalence of 8.6% [2]. AD is, inter alia, characterized by larger amounts or longer periods of alcohol intake than intended, withdrawal when stopping, craving, and continued alcohol intake despite social, interpersonal, or health problems [1]. Especially, the reported alcohol craving, which describes an uncontrollable desire to take a substance, is one of the central aspects in the development and maintenance of alcohol dependence and is often jointly responsible for relapses [3]. Reducing this subjective craving therefore is an important goal to increase the rates of abstinence by preventing relapses, along with negative impacts on health and quality of life in AD patients.

At the same time, it can be observed clinically that patients try to reduce their cravings by the intake of higher amounts of high-carbohydrate food, for example, sweets. This observation is supported by study results: There is a link between glucose levels and alcohol-seeking behavior [4], and the sugar craving increased in 14 out of 35 patients during the withdrawal treatment [5]. Following these results, the intake of high-carbohydrate food seems to reduce alcohol craving, and it stands to reason that appetite-regulating hormones play an essential role in the underlying mechanism of AD. Particularly with regard to craving and relapse, acetylated ghrelin and insulin take high priority as evidence suggests that those hormones influence craving in early abstinence [6, 7].

Ghrelin is mainly produced as pre-pro-ghrelin in neuroendocrine cells of the gastric mucosa. In a two-stage activation process, pre-pro-ghrelin is shortened to pro-ghrelin and then forms the 28-amino-acid peptide ghrelin through the process of proteolytic cleavage [8]. Only after post-translational acetylation, ghrelin becomes functional [9]. The release of ghrelin and therefore the increase of ghrelin plasma concentration promote feelings of hunger. When saturated, the plasma concentration of ghrelin decreases [10–12].

Meanwhile, there is evidence that ghrelin also plays a role in the reward system, more precisely in the mesolimbic dopaminergic pathways and therefore in the development and maintenance of AD [13]. Dopaminergic neurons project from the ventral tegmental area (VTA) to the nucleus accumbens (NAc), the ventral striatum (VS), and afterwards into the prefrontal cortex (PFC) [14]. When dopamine release increases, people focus more on stimuli like food or alcohol and less on irrelevant environmental stimuli [15]. The acetylated form of ghrelin stimulates the activity of these dopaminergic neurons and therefore the activity of the mesolimbic reward system [16]. Consequently, higher concentrations of ghrelin go along with the higher activity of dopaminergic neurons and an increased focus on alcohol-related cues.

There is increasing evidence emphasizing the positive association of acetylated ghrelin and alcohol craving in early abstinence (for a review, see [17]). A preliminary study of our own working group including 41 AD patients during early abstinence confirms a positive association between acetylated ghrelin and subjective craving, with the effect mediated by cue-induced mesolimbic brain response [17]. A recent study [6] proves a significant increase in acetylated ghrelin plasma concentration in early abstinence, supporting the evidence of a further study of our working group, postulating that the acetylated ghrelin plasma concentration increases significantly during early abstinence [18]. The administration of ghrelin, for example, by intravenous injection, leads to significant higher cravings than the administration of a placebo [19]. The administration of a ghrelin receptor antagonist has the opposite effect: Preclinical animal intervention studies suggest that alcohol preference and consumption decrease after the administration of ghrelin receptor antagonists [20–25].

Preliminary studies could show positive associations between plasma concentration of acetylated ghrelin and brain activation in the striatum, as well as in the insula [17, 26]. Additionally, some studies postulate that intravenous administration of ghrelin leads to an increase in alcohol-associated brain activation in fMRI [27]. However, the systematic administration of a ghrelin receptor antagonist prevents ghrelin from increasing the release of dopamine in VTA and NAc and therefore reduces alcohol-related cue reactivity [25].

Besides ghrelin, evidence suggests that insulin also influences cravings in early abstinence, although there are few randomized controlled studies to date. Nevertheless, this assumption seems plausible, as studies show the negative correlation between ghrelin and insulin plasma levels in healthy participants [28, 29].

Insulin is produced in the pancreas as proinsulin and then cleavaged to a 51-amino-acid peptide and a C-peptide. The peptide hormone insulin is also part of food regulation, and its main function is to transport glucose from the blood into cells [12]. The distribution of insulin leads to a decrease in blood sugar levels and is therefore indispensable for the use of glucose as an energy source after meals [30].

Insulin receptors are located in the dopaminergic neurons of VTA, the striatum (especially NAc and substantia nigra), and the hypothalamus and amygdala [31]. Since these regions are of particular relevance in the development of addiction [32], it is assumed that besides ghrelin, insulin also plays a role in the development and maintenance of alcohol dependence [33]. The release of insulin inhibits the excitatory synapses onto VTA dopamine neurons and therefore the activity of dopaminergic neurons [14]. Consequently, contrary to the findings on the role of ghrelin, higher concentrations of insulin go along with the lower activity of dopaminergic neurons and therefore lower subjective craving.

Supporting the theoretical assumptions, craving for nicotine decreased significantly in smoking abstinent patients after intranasal insulin administration compared to placebo [34]. Similar results can be assumed for AD patients due to the shared underlying processes of nicotine and alcohol use [35].

Regarding the evidence for the role of insulin in mesolimbic cue reactivity, insulin induces long-term depression (LTD) of excitatory synapses onto VTA dopamine neurons and therefore reduces the focus of food-related cues in the mouse brain [36]. A preclinical animal study could show that the administration of insulin into the VTA reduces cocaine-induced dopamine release in NAc [7]. Based on these results, similar results can be assumed in people suffering from AD due to the shared underlying processes of dopamine release in the mesolimbic reward system of cocaine and alcohol [37] even if the clinical findings are not yet sufficient.

Regarding the presented evidence, innovative treatment approaches could include the induction of changes in the hormones mentioned and thereby changes in craving and mesolimbic cue reactivity [38].

However, long-term administration of a ghrelin receptor antagonist or insulin injections is not possible due to the expected side effects on weight regulation, with the risk of pronounced weight loss. However, the induction of changes in plasma concentrations of acetylated ghrelin or insulin can be a short-term treatment option to reduce acute craving when needed, consistent with clinical observations. To achieve this aim, ghrelin and insulin plasma concentrations can physiologically be affected by the ingestion of foods or a solution containing glucose as examined in the present trial. Alternative ways of physiologically influencing these plasma concentrations are gastric distension [39] and lipid intake [40], which are particularly relevant if there are contraindications to glucose intake (e.g., a disorder of glucose metabolism such as diabetes mellitus). A randomized clinical preliminary study proves that forced oral water intake (1000 ml in 10 min) leads to gastric distension, following decreased acetylated ghrelin plasma levels and therefore a reduction of subjective alcohol craving during early alcohol abstinence in patients suffering an alcohol dependence [39]. Following these promising results, the present paper describes the study protocol and presents the methods of a randomized, placebo-controlled, double-blind crossover study, investigating the effects of a forced oral glucose intake on subjective craving and mesolimbic cue reactivity. The study aims to provide evidence for the physiological basis of the clinically observed benefits of glucose intake in early abstinence in AD patients, based on the theoretical assumptions presented above. The paper follows the SPIRIT guidelines for the study protocols [41].

## Methods

### Clinical trial design

The G-CuP (English: Glucose - Craving and Peptides) trial is designed as a monocenter randomized, double-blind, placebo-controlled, crossover trial examining two treatment arms. *N* = 108 individuals suffering from an AD and receiving a standard in-patient treatment (including withdrawal treatment, psychotherapeutic individual and group sessions, skills training, relaxation exercises, sports, occupational therapy, sociotherapy, pharmacotherapies if required, and aftercare planning) will be examined in the study.

Following the crossover design, participants will undergo both investigation conditions (glucose intake and placebo intake) on two measuring days within the first 7 to 21 days of abstinence. For each participant, the trial consists of a screening visit S0 (− 7 to − 1 day before T1) and two treatment visits T1 (day 0) and T2 (+ 3 to + 7 days). The crossover design was chosen because it is considered the gold standard for clinical trials. Fewer study participants are needed, even small effects of the intervention can be statistically proven and none of the participants is deprived of the intervention. The purpose of conducting a double-blind study is to prevent bias in the study results on the part of both the study investigators and the subjects.

**Fig. 1:**
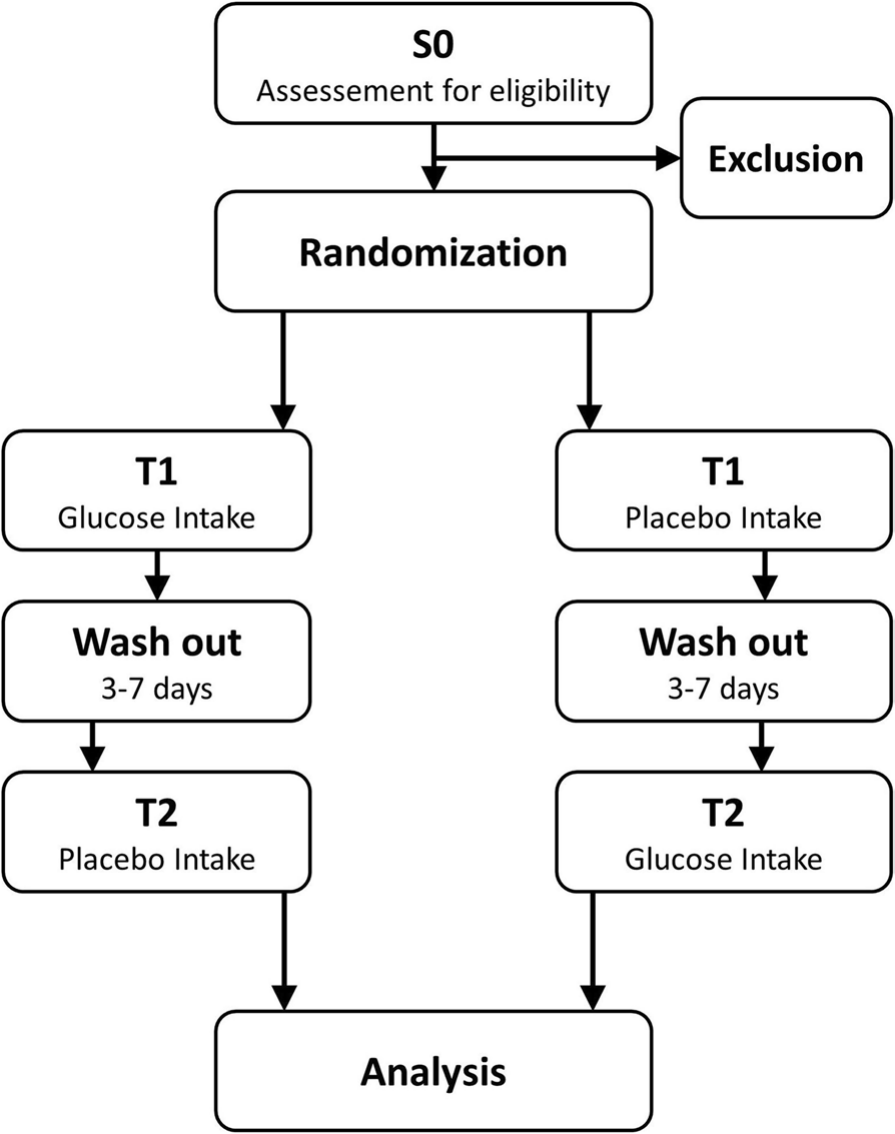
Clinical trial design

The overall flowchart of the clinical trial design is shown in Fig. 1.

### Study objectives

The main aim of the present trial is to assess the potential effects of a forced oral glucose intake on changes of acute subjective craving within 70 (without fMRI measurement) to 100 min (including fMRI measurement) after the intake, which are modulated by changes in plasma concentration of acetylated ghrelin and insulin compared to a placebo intake (primary outcome).

Additionally, the study examines the effects of a forced oral glucose intake on mesolimbic cue reactivity in fMRI when confronted with alcohol-related cues, more precisely the neural brain activation (blood oxygenated level dependent (BOLD)) in the mesolimbic reward system during the presentation of alcohol cues and neutral cues compared to a placebo intake (secondary outcome).

Based on the presented state of research to date, the following hypotheses are proposed:

1. A forced oral glucose intake leads to a reduction of subjective alcohol craving which is modulated by a reduction of acetylated ghrelin plasma concentration and simultaneously an increase of insulin plasma concentration.
2. A forced oral glucose intake leads to a reduction of activation in the mesolimbic reward system after a confrontation with alcohol-related stimuli (cue reactivity) in fMRI, modulated by a reduction of acetylated ghrelin plasma concentration and simultaneously an increase of insulin plasma concentration.
3. There is a positive association between the reduction of subjective alcohol craving, the reduction of cue reactivity in mesolimbic reward system, and the reduction of acetylated ghrelin plasma concentration such as the increase of insulin plasma concentration.

### Study population

Recruitment should take place in German hospitals offering alcohol withdrawal treatment, to detect intervention-related differences in craving and mesolimbic cue activity in early abstinent patients. Therefore, recruitment takes place at the Department of Addictive Behavior and Addiction Medicine at the Central Institute of Mental Health, Mannheim (CIMH), and a cooperating department of the Psychiatric Center North Baden, Wiesloch (PZN), as part of the Feuerlein Center on Translational Addiction Medicine (Feuerlein CTS). Both clinics are psychiatric hospitals meeting the highest standards of treatment and research, containing specific departments for addictive behavior and addiction medicine, including specific wards for alcohol withdrawal treatment.

The following inclusion and exclusion criteria were chosen based on preliminary studies [17, 18, 26, 39] and ensure a representative group of alcohol-dependent patients.

#### Inclusion criteria

Male and female patients from the in-patient unit of CIMH and PZN aged 18 to 65 years are examined in the study. They have to meet the criteria for alcohol dependence according to ICD-10 [1], as well as have completed their medically supervised detoxification supported by benzodiazepines for at least 5, and a maximum of 21 days. Participants must be able to take part in a supervised alcohol exposure session and a fMRI measurement. All participants must give written informed consent before being included.

#### Exclusion criteria

The study excludes patients fulfilling any axis-I psychiatric disorder according to ICD-10, besides alcohol dependence, except for nicotine abuse or dependence, mild or moderate depressive episodes, and recurrent depressive disorder, current episode mild or moderate as well as adjustment disorders. Furthermore, a positive urine drug screening; the current intake of psychotropic medications, except for antidepressants and low potency neuroleptics; or suicidal tendencies result in exclusion. The trial does not exclude participants suffering with the abovementioned comorbidities and medication because they often co-occur with alcohol dependence and are typical for a representative group of alcohol-dependent patients [42].

Additionally, patients suffering from carbohydrate metabolism disorders (e.g., diabetes mellitus) are excluded, due to the expected effects of glucose on blood sugar levels. Patients who have an allergy to bitter almond aroma or sodium cyclamate due to the ingredients of the glucose and placebo solutions are also excluded (for details see the “Intervention” section). Further exclusion criteria are meeting any contraindications for conducting a fMRI scan (e.g., tattoos, metal implants, pregnancy, or claustrophobia) and the participation in another clinical trial taking place simultaneously.

Screening for the inclusion and exclusion criteria includes the Structured Clinical Interview for DSM-V (SCID-5-CV) that presents high reliability and specificity, as well as validity, and is well-established in clinical and science practice [43]. The SCID-5-CV is conducted by a skilled psychologist. Additionally, a medical check is conducted by a physician specially trained for the study to control for contraindications for study participations, especially fMRI exclusion criteria. On the screening day, a drug and a pregnancy test are mandatory.

### Recruitment

Recruitment of participants is carried out in the in-patient units of CIMH and the cooperating hospital PZN. The recruitment process started in August 2020 and will probably be completed in January 2023. Recruitment is conducted by psychologists and physicians specially trained for the study. The participants’ recruitment process is supported by flyers, which raise awareness for the study.

### Organization and monitoring of recruitment process

The study is coordinated at CIMH. The principal investigator Dr. Anne Koopmann and the study lead Lea Wetzel are responsible for the study organization. They also organize the recruitment, contact the study participants, and coordinate the collaboration of the study staff. Monthly meetings of the entire study team are held for quality assurance and process optimization.

### Randomization and blinding

According to the crossover design, all subjects go through both investigation conditions, the glucose and the placebo condition. Participants are allocated randomly to treatment order (glucose-placebo/placebo-glucose) with equal probability (1:1 ratio) by an independent pharmacy employee at the pharmacy of University Medical Center Heidelberg, using the RITA version 1.31 software (Randomization In Treatment Arms, Evidat, Germany) and a block randomization scheme with block sizes of 6. All participants will receive a unique anonymous identifying number used for the study documentation to ensure anonymity.

The study is conducted under double-blind conditions, so that neither the study staff nor the participants are aware of the assignment. Only the study manager, who is not directly involved in data collection, knows the order of conditions. The blinding will be maintained until all 108 subjects have completed the study and the database is locked. In the case of a medical emergency that requires knowledge of the treatment condition, the study staff can open the sealed envelope to unblind the study condition.

### Intervention

#### Glucose vs. placebo intake

On each intervention day, participants receive either a highly concentrated glucose solution (80 g glucose solved in 200 ml of water) or a placebo solution (1.6 g sodium cyclamate solved in 200 ml of water). Both solutions contain an equivalent degree of sweetness, and possible differences in taste are balanced by bitter almond flavoring. The amount of liquid was chosen in order to prevent any volume effects on the plasma concentrations of ghrelin and therefore the craving or the cue reactivity [39]. The dry matter is delivered by a pharmacy of the University Medical Center Heidelberg and dissolved in water on the examination day by the trained study manager. The solution must be consumed orally by the participant within 10 min.

In accordance with previous trials [17, 26, 39, 44], all participants will receive treatment as usual during the examination period. Participants should continue fixed-dose medication intake as usual. However, if it is anticipated that the participant will need new medication such as anxiolytics or relapse prevention medication during the intervention phase, they will be ineligible for entry into the study or need to be excluded immediately, if they have already entered the study. All participants will remain in the in-patient units of CIMH or PZN for at least 24 h after the last examination day to monitor any possible side effects such as increased craving.

#### Alcohol cue exposure session

During the alcohol cue exposure session, participants will be exposed to their favorite and most consumed alcoholic beverage to increase the participant’s subjective craving. The alcohol cue exposure was established and validated in previous studies; for details, see Koopmann et al. [45]. The exposure session is carried out by clinically trained staff and supervised in order to ensure the standardization of the sessions.

### Data collection and outcome measures

**Fig. 2:**
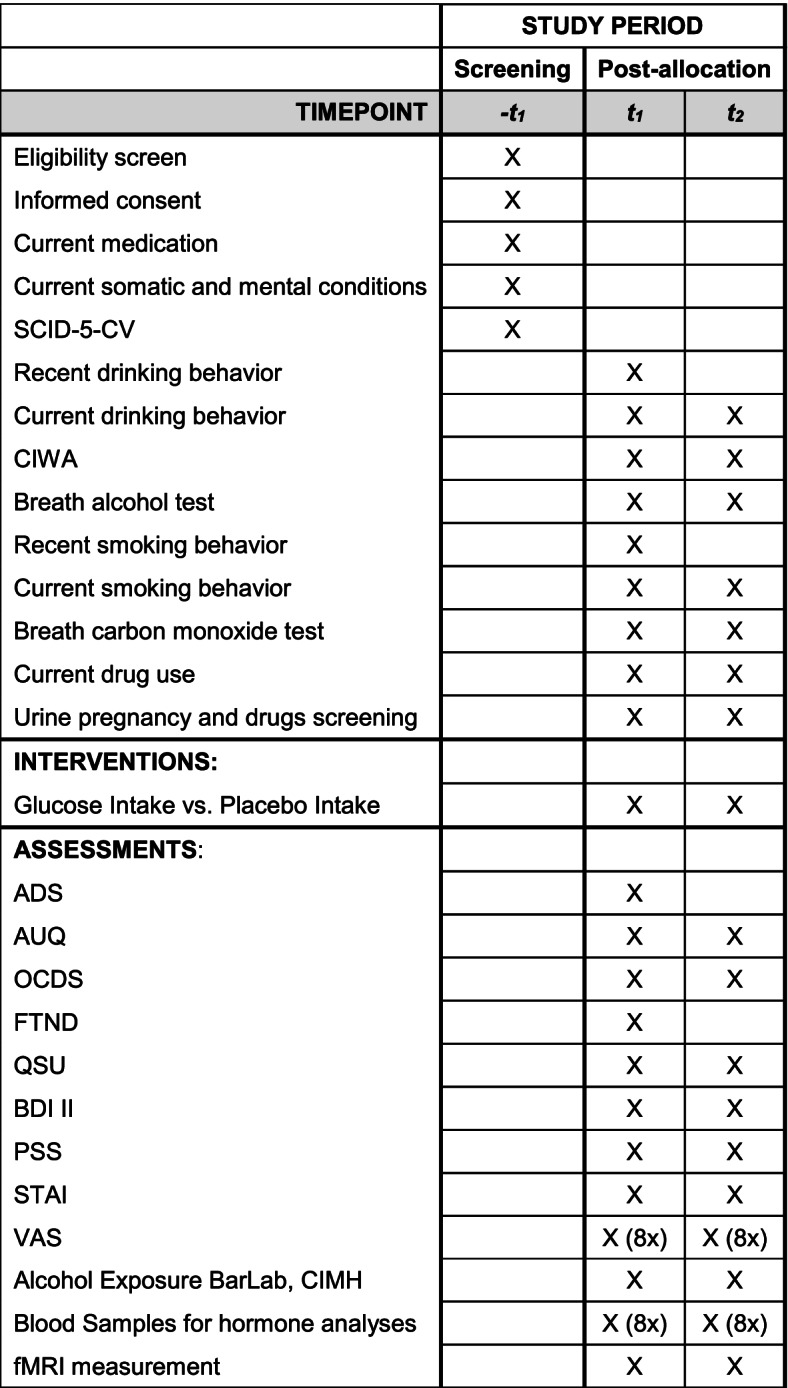
SPIRIT figure of the G-CuP study protocol

The trial design contains the assessment of demographic and psychometric data, a craving self-report, an alcohol cue exposure, a fMRI measurement, and laboratory blood tests (see Fig. 2 [41]).

Changes in craving within 70 (no fMRI measurement) and 100 min (including fMRI measurement) after the glucose intake, as a primary outcome, will be measured by the Alcohol Urge Questionnaire (AUQ) at the beginning of each measuring day and visual analog scales (VAS) at eight time points during the trial. The AUQ consists of eight statements about the participants’ feelings and thoughts about alcohol consumption. The VAS includes the question “How high is your perceived craving for alcohol at this moment?” answered on an 11-item Likert scale ranging from “0 = no craving” to “10 = highest craving.”

fMRI measurements are conducted to examine the mesolimbic cue reactivity as a secondary outcome. The measurements are first conducted at a 3-T MAGNETOM Trio whole-body tomograph (Siemens, Erlangen, Germany) and after an upgrade in December 2021 at a 3-T MAGNETOM Prisma whole-body tomograph (Siemens, Erlangen, Germany) at the CIMH. Besides fieldmap and localizer, a functional resting state scan with eyes closed and a T1-weighted anatomical MPRAGE scan, the scan sessions contain the validated paradigms ALCUE [46] and NiCUEtin [47].

The nicotine-related mesolimbic stimulus reactivity is examined because of the high comorbidity between AUD and nicotine consumption and possible additive rewarding effects by combined consumption [48]. Besides, participants are asked to rate their current alcohol or nicotine craving on a visual analog scale during the paradigms.

Additionally, various self-assessment questionnaires (see Table 1) are assessed to detect possible covariates influencing the relationship of acetylated ghrelin and insulin plasma concentrations, subjective craving, and mesolimbic cue reactivity.

**Table 1:** Questionnaires and psychometric scales incorporated in the study

Measure	Description
Alcohol Dependence Scale (ADS; [49])	Severity of the participant’s dependence on alcohol
Alcohol Urge Questionnaire (AUQ; [50])	Subjective alcohol craving
Obsessive Compulsive Drinking Scale (OCDS; [51])	Obsessive thoughts about alcohol use and compulsive behaviors related to drinking
Fagerström Test for Nicotine Dependence (FTND; [52])	Intensity of physical addiction to nicotine
Questionnaire of Smoking Urges (QSU; [53])	Subjective nicotine craving
Beck Depression Inventory (BDI II; [54])	Depressive symptoms
Perceived Stress Scale (PSS; [55])	Subjective stress
State and Trait Anxiety Inventory (STAI; [56])	Anxiety as a personality and at the moment

To observe the changes in plasma concentrations of acetylated and total ghrelin such as insulin, in both study conditions, eight blood samples (9 ml) of each participant are taken within 2 h (approximately every 10th minute). All samples are obtained by an indwelling venous cannula (Vasofix® Braunüle® 1.30 × 45 mm G 18 green, FEP), anticoagulated with sodium EDTA (1 mg/ml whole blood, Sarstedt, S-Monovette® 9 ml, serum with coagulation activator, 92 × 16 mm) and then immediately cooled on ice. Plasma is processed by the in-house biobank, more precisely separated in a centrifuge at 4000*g* for 10 min temperated at 4 °C. Aliquots are then filled into 2-mL Protein LoBind tubes and immediately frozen and stored at − 80 °C until the time of analysis. Subsequently, hormonal analyses will be performed at the Molecular Neurobiology Laboratory of the Department of Psychiatry and Psychotherapy at the University Medical Center Erlangen when data of all included patients have been collected.

### Sample size

Sample size calculation was carried out, based on the changes in subjective craving after forced oral glucose intake as a primary outcome. Based on the pilot study [39], the lower confidence limit for the 95% confidence level (one-sided) was calculated as the base effect size of 0.38. Transferred to the scale for crossover design, this corresponds to an effect size of 0.76. Applied to an unpaired *t*-test with a significance level of 5% (two-sided) and a power of 90%, a *N*_0_ of 76 results. Assuming a dropout rate of 30% during the study, *N* = 108 patients should be included in the experiment. It is reasonable to assume that the number of cases calculated for the primary outcome is also sufficient for the fMRI data, as previous studies showed that effect sizes of brain activation changes are significantly higher than effect sizes of changes in behavioral studies [57].

### Statistical methods

#### Statistical analysis of anamnestic, psychometric, and hormone data

To analyze the anamnestic, psychometric, and hormone data, the statistic software IBM SPSS (Statistics – Advanced Statistics for Windows, version 26, IBM Corp., Armonk, NY) will be used. Descriptive data will be presented as statistical average (mean values) with standard deviations. To examine the difference in craving after exposition as a primary outcome, an unpaired *t*-test will be conducted. To determine the changes in craving and ghrelin and insulin plasma concentrations over the time during the measuring day and between the measuring days as the primary outcome, mixed linear models incorporating measurement repetition and crossover design will be calculated. Associations between changes in plasma concentrations of ghrelin and insulin and craving will be computed using the Pearson correlation coefficient.

#### Statistical analysis of fMRI data

To analyze the fMRI data, SPM12 (Wellcome Centre for Human Neuroimaging, Institute of Neurology, University College London, UK) running under Matlab R2020a (MathWorks, Natick, USA) will be used. Pre-processing will include motion correction, normalization to the Montreal Neurological Institute (MNI) template, and a spatial smoothing using an isotropic Gaussian kernel of 8 mm full width at half maximum (FWHM). Afterwards, first-level analyses (within-subject) modeling the experimental conditions (alcohol-related stimuli, neutral stimuli, rating phase via VAS) will be conducted for each participant in a general linear model. The resulting contrast images will be imputed into second-level analyses (between-subject) conducting induced brain activity regarding the effects of group and time using paired *t*-tests. To control for multiple statistical testing, the probability of a family-wise error (FWE) will be set to pFWE < 0.05. Associations between ghrelin and insulin plasma concentrations and cue-induced brain activation will be calculated using multiple regression analyses.

#### Risk assessment, risk management, and missing data

Due to the chosen study design (crossover design with two investigation conditions including two fMRI measurements), there is a risk that participants abandon the trial early after the first measuring day. In case of a dropout rate higher than 50%, only data of the first examination day of each case will be evaluated. Due to the randomization, the analysis of data of the intervention group on the first day and data of the control group on the first day should lead to comparable study populations. Due to the planned study course, there is a risk that included participants abandon the examination prematurely during the measuring day. Only values for measurement points will be included, where at least 50% of participants are still participating.

### Confidentiality and data quality

To ensure data quality, questionnaires are filled out electronically and stored automatically in the study database. Other data, such as any deviations from the study protocol, will initially be documented on paper-based documents. Data will then be transferred promptly into a spreadsheet stored on a password-protected data drive. Data entry and management will be completed by one of the study coordinators and double-checked by at least one other study employee. All source documents and data sheets used in the clinical trial will not include any patient identifying information. Study data will be stored password-locked on servers separated from the personal information of the participants. All information pertaining to the study will be stored for 10 years, in line with the requirements of the local ethics committee.

## Discussion

Alcohol dependence is a disorder that is often associated with a long history of illness, physical sequelae, and high relapse rates after treatment. In particular, the craving of the subjects leads to these relapses [3]. New medications or interventions that have been tried in recent years can unfortunately only have a small impact on relapse rates. In view of the encouraging results of another study by our research group, which showed a reduction in craving after forced volume intake moderated by the hormone ghrelin [39], the use of a glucose solution against the background of similar mechanisms is very promising for the short-term reduction of craving in alcohol-dependent patients.

The present study, through its crossover design, will allow us to assess the overall efficacy of a glucose solution in reducing patients’ craving for alcohol. In addition, we will be able to assess whether forced glucose intake also leads to a reduction in mesolimbic cue reactivity when alcohol-associated stimuli are presented. The intervention being evaluated in this randomized controlled trial is designed to be accessible and easy to use for the alcohol-dependent patients. If the results are positive, glucose solution ingestion could be self-administered by patients as an acute, fast-acting, easy-to-use, and cost-effective technique to rapidly reduce subjective alcohol craving in acute cases and thus prevent relapses.

### Trial status

Protocol version 1, 06/01/2022. The trial is ongoing, and participants are being actively recruited. Recruitment began in August 2020 and will be completed approximately January 2023.

## Data Availability

Individual de-identified data will be made public; in particular, individual participant data underlying the study outcomes will be provided with a corresponding data dictionary. Secondary outcome data will be made available on an aggregated group level in online repositories. Individual data will be shared with researchers who submit a methodologically sound proposal (sent to the principal investigator of the trial).
