# Correction: G-CuP: the effect of a forced oral glucose intake on alcohol craving and mesolimbic cue reactivity in alcohol dependence—study protocol of a randomized, double-blind, placebo-controlled crossover study

**DOI:** 10.1186/s13063-022-06888-4

**Published:** 2022-11-10

**Authors:** Lea Wetzel, Madeleine Pourbaix, Alisa Riegler, Anna-Maria Pfeifer, Iris Reinhard, Sabine Hofmann, Sabine Vollstädt-Klein, Falk Kiefer, Wolfgang Sommer, Jan Malte Bumb, Patrick Bach, Anne Koopmann

**Affiliations:** 1 Medical Faculty Mannheim, Heidelberg University, Heidelberg, Germany; 2 Department of Addictive Behavior and Addiction Medicine, Central Institute of Mental Health (CIMH), Medical Faculty Mannheim/ Heidelberg University, Mannheim, Germany; 3 Feuerlein Centre on Translational Addiction Medicine (FCTS), University of Heidelberg, Heidelberg, Germany; 4 Department of Biosta- tistics, Central Institute of Mental Health (CIMH), Medical Faculty Mannheim/ Heidelberg University, Mannheim, Germany; 5 Institute of Psychopharmacol- ogy, Central Institute of Mental Health (CIMH), Medical Faculty Mannheim/ Heidelberg University, Mannheim, Germany

**Correction: Trials 23, 693 (2022)**

**https://doi.org/10.1186/s13063-022-06626-w**

Following publication of the original article [1], the authors identified an error in the author name of Patrick Bach.

The incorrect author name is: Patrick Back

The correct author name is: Patrick Bach

The author group has been updated above and the original article [1] has been corrected.
